# Genome-Wide Analysis and Expression Profiling of Glutathione Reductase Gene Family in Oat (*Avena sativa*) Indicate Their Responses to Abiotic Stress during Seed Imbibition

**DOI:** 10.3390/ijms231911650

**Published:** 2022-10-01

**Authors:** Ming Sun, Shoujiang Sun, Zhicheng Jia, Wen Ma, Chunli Mao, Chengming Ou, Juan Wang, Han Zhang, Liu Hong, Manli Li, Shangang Jia, Peisheng Mao

**Affiliations:** Forage Seed Laboratory, College of Grassland Science and Technology, China Agricultural University, Beijing 100193, China

**Keywords:** *A. sativa*, glutathione reductase, abiotic stress, seed imbibition, qPCR

## Abstract

Abiotic stress disturbs plant cellular redox homeostasis, inhibiting seed germination and plant growth. This is a crucial limitation to crop yield. Glutathione reductase (GR) is an important component of the ascorbate-glutathione (AsA-GSH) cycle which is involved in multiple plant metabolic processes. In the present study, GRs in *A. sativa* (*AsGRs*) were selected to explore their molecular characterization, phylogenetic relationship, and RNA expression changes during seed imbibition under abiotic stress. Seven *AsGR* genes were identified and mapped on six chromosomes of A, C, and D subgenomes. Phylogenetic analysis and subcellular localization of AsGR proteins divided them into two sub-families, AsGR1 and AsGR2, which were predicted to be mainly located in cytoplasm, mitochondrion, and chloroplast. *Cis*-elements relevant to stress and hormone responses are distributed in promoter regions of *AsGRs*. Tissue-specific expression profiling showed that *AsGR1* genes were highly expressed in roots, leaves, and seeds, while *AsGR2* genes were highly expressed in leaves and seeds. Both *AsGR1* and *AsGR2* genes showed a decreasing-increasing expression trend during seed germination under non-stress conditions. In addition, their responses to drought, salt, cold, copper, H_2_O_2_, and ageing treatments were quite different during seed imbibition. Among the seven *AsGR* genes, *AsGR1-A, AsGR1-C, AsGR2-A,* and *AsGR2-D* responded more significantly, especially under drought, ageing, and H_2_O_2_ stress. This study has laid the ground for the functional characterization of GR and the improvement of oat stress tolerance and seed vigor.

## 1. Introduction

Reactive oxygen species (ROS) are key regulators of plant growth and development throughout the whole plant life cycle [1,2]. Without stresses, ROS in the organism maintain the dynamic equilibrium by various antioxidative defense mechanisms [3,4]. The equilibrium of ROS-producing and ROS-scavenging can be changed under stressful conditions, which thus leads to the excessive accumulation of ROS, lipid peroxidation, protein oxidation, and nucleic acid damage [4]. Recent studies have substantiated that ROS play a pivotal role in the regulation of seed germination, dormancy, and longevity [1,2,5]. However, adverse seed storage and germination conditions often lead to ROS accumulation, and inhibit germination and normal seedling establishment. The major sources of ROS production in plants are chloroplasts, mitochondria, peroxisome, apoplast, etc. [3]. In orthodox seeds, the mitochondrion is the main source of ROS (especially in seed germination) [6]. Therefore, alleviating the excessive accumulation of ROS and maintaining the dynamic balance of ROS are key to coping with the adverse environment.

In plants, the ascorbate-glutathione (AsA-GSH) cycle as an antioxidant system, which involved enzymatic and non-enzymatic antioxidants, plays a vital role in detoxifying ROS [7]. Glutathione (GSH; c-glutamyl-cysteinyl-glycine), an omnipresent low-molecular-weight thiol compound, is a strong non-enzymatic antioxidant and an essential metabolite with multifunction in plants [8,9]. It participates in various biological processes, including antioxidant defense, redox signaling, xenobiotics detoxification, etc. [8,9]. Due to the nucleophilic nature of their cysteine residues, GSH can be converted to oxidized glutathione disulfide (GSSG). Typically, the intracellular GSH concentration is kept at the millimole levels, and up to 90% GSH is in its reduced form under normal conditions [8]. Glutathione reductase (GR, EC 1.6.4.2) belongs to the NADPH-dependent oxidoreductase group of flavoproteins and plays an important role in catalyzing the reduction of GSSG to GSH using NADPH as electron donor in eukaryotes and prokaryotes [7,10]. The redox balance of the GSH/GSSG couple is essential for maintaining seed vigor and normal germination, and the increase of endogenous and exogenous GSH is beneficial for seed vigor improvement under stress [11,12,13]. The expression levels of *GR* genes can be significantly induced by unfavorable conditions. The deletion or down-regulation of *GR* genes would lead to the decrease of seed vigor [14]. Moreover, GR activity increases during germination both in embryo and endosperm. These suggest that GSH and GR play a considerable role in the maintenance of the intracellular redox balance and biological functions.

In plants, GRs are mainly localized in chloroplast, mitochondria, and cytosol [10]. Previous works have classified GR proteins into two clades of GR1 and GR2 on the basis of the presence of N-terminal extension. GR1 is a shorter cytosolic enzyme, and GR2 representing the elongated organellar proteins can target the GR proteins in both mitochondria and chloroplast [15]. NADPH binding domain, FAD binding domain, and the interface domain binding the two subunits of GR proteins are identified in GR proteins. GRs have been suggested to play a central role in maintaining the reduced GSH pool and improving abiotic and biotic stress resistance [10,16,17]. The localizations where GR is active are also the sources of ROS, such as chloroplasts and mitochondria. Oxidative stress caused by drought, temperature, heavy metals, salinity, and hydrogen peroxide can induce changes of *GR* gene expression and GR enzyme activity [10,18,19]. Overexpression of GRs improved the stress resistance in transgenic plants, and the deletion of GRs led to the decrease of antioxidant capacity [14,20,21,22].

The GR protein encoded by a small gene family has been described in many crops and model plants, such as *Arabidopsis thaliana*, *Brassica juncea*, *Brassica rapa*, *Oryza sativa*, *Populus trichocarpa*, *Aegilops tauschii*, and *Triticum aestivum* [15,17,23,24]. The genome-wide identification of *GRs*, and characterizing its structure and function, would be useful for future functional and mechanism studies on GR proteins. In addition, RNA-seq and quantitative real-time PCR (qPCR) analysis of *GR* genes indicated that they strongly responded to abiotic and biotic stresses and plant hormones [17,18]. Typically, two or three GRs are found in the above reported species. Recently, seven *GR* genes were identified in the allohexaploid genome (AABBDD) of bread wheat [24]. Oats (*Avena sativa*) are also an allohexaploid species (AACCDD, 2 n = 6 x = 42) and rank sixth in world cereal production due to high nutritional and medicinal value and broad distributions [25]. The identification of essential gene families, especially antioxidant gene families, has not been carried out in oats, as the chromosome-scale reference genome was recently available (https://wheat.pw.usda.gov/GG3/graingenes-data-downloads, accessed on 24 November 2021) [26,27]. In addition, the central roles of GSH and GRs in seed vigor maintenance and seed germination have been demonstrated, but the divergent roles of different GR isoforms in seed germination under stress have not been reported. The aim of this study was to identify *A. sativa GR* genes in the whole genome level, and perform analysis of gene and protein structure, in silico subcellular localization prediction, and biochemical characterization. Moreover, the relative expressions of these members in response to the treatments of drought, salt, cold, CuSO_4_, H_2_O_2_, and ageing were compared by qPCR, in seed imbibition. This work can be utilized in future genetic improvement of *A. sativa* for stress tolerance and seed vigor.

## 2. Results

### 2.1. Identification and Chromosomal Mapping of GR Genes

**Figure 1:**
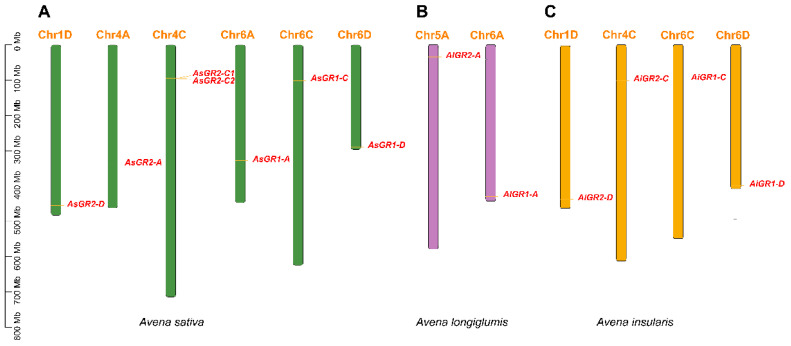
Chromosomal mapping of *GR* genes in *A. sativa* (**A**), *A. longiglumis* (**B**), and *A. insularis* (**C**). The chromosome number is denoted on the top of each chromosome. The scale is marked in megabases (Mb).

A total of thirteen *GR* genes were identified in *A. sativa* and its possible ancestors, namely *A. longiglumis* and *A. insularis*. The diploid *A. longiglumis* (AA) consisted of two *GR* genes which were distributed in chromosome 5A and 6A, respectively (Figure 1B). Four *GR* genes were identified from tetraploid *A. insularis* (CCDD), and they were distributed in chromosomes of 1D, 4C, 6C and 6D (Figure 1C). However, seven *AsGR* genes located at chromosomes of 1D, 4A, 4C, 6A, 6C and 6D were identified from allohexaploid *A. sativa* (Figure 1A). According to homologous relationships with the two *Arabidopsis* GR proteins and their sub-genomic locations, *A. sativa GR* genes were clustered into two homologous groups *AsGR1* and *AsGR2*, and named as *AsGR1-A*, *AsGR1-C*, *AsGR1-D*, *AsGR2-A*, *AsGR2-C1*, *AsGR2-C2*, and *AsGR2-D*. *A. longiglumis GR* genes were named as *AlGR1-A* and *AlGR2-A*, and *A. insularis GR* genes were named as *AiGR1-C, AiGR1-D, AiGR2-C*, and *AiGR2-D*.

Chromosome mapping results showed the number of *AsGR* genes were one more than the six ones by combining its diploid (*A. longiglumis*) and tetraploid (*A. insularis*) progenitors. Chromosome 4C of *A. insularis* had a single *GR* gene, while chromosome 4C of *A. sativa* consisted of two *AsGR* genes, which revealed the gain of one more *GR* gene during evolution. Both chromosome 5 and 6 of AA progenitor had one *GR* gene, respectively, while there is no *GR* gene in chromosome 5A of *A. sativa.* Instead, chromosome 4A gained a *AsGR* gene. This indicated an occurrence of a recombination between different chromosomes during evolution. Moreover, most of the *AsGR* genes are distributed at the ends of chromosomes, which also suggests their potential translocation between different chromosomes. 

### 2.2. Physico-Chemical Properties of AsGR Family

We looked into the physico-chemical properties of AsGR proteins, including the amino acid composition, molecular weight, theoretical isoelectric point (pI), instability index, and negatively and positively charged residues. The molecular weight of AsGRs was ranging from 34.718 to 93.097 kDa, which is in accordance with the amino acid length from 321 (AsGR2-C2) to 853 (AsGR1-D). All three AsGR1 members showed slightly acidic pI, while AsGR2 members showed divergent pI. AsGR2-C2 showed acidic pI (5.34), AsGR2-C1 and AsGR2-D showed nearly neutral pI (6.93), and AsGR2-A had alkalic pI (9.59). The instability index prediction showed that AsGR1 proteins and AsGR2-C2 were stable (Instability index < 40), while AsGR2-C1, AsGR2-D, and AsGR2-A were classified as unstable ones with high instability index. Additionally, almost all of the AsGR proteins were found to be rich in negatively charged residues, except for AsGR2-A (Table 1).

### 2.3. Phylogenetic Analysis and In Silico Subcellular Localization of GR Proteins

**Figure 2:**
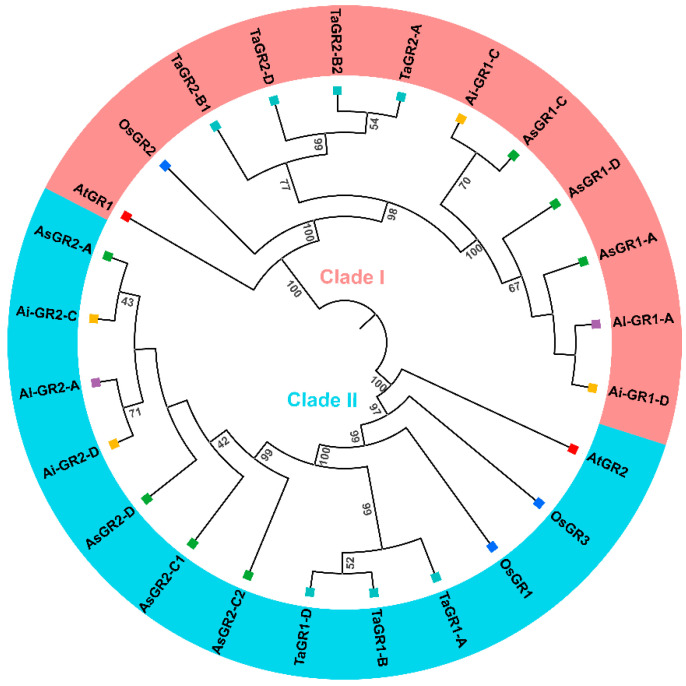
Phylogenetic relationships of GR proteins. The phylogenetic tree based on GR sequences from *A. sativa* (green)*, A. longiglumis* (purple), *A. insularis* (orange) *Arabidopsis* (red)*,* rice (blue), and bread wheat (turquoise) was constructed using MEGA v6 with the neighbor-joining (NJ) method, and the bootstrap test replicate was set as 10,000 times.

To understand the phylogenetic relationship and classification of GR proteins, we constructed a phylogenetic tree with the protein sequences of GRs from *A. sativa*, *A. longiglumis*, and *A. insularis*, as well as known GRs classified from *Arabidopsis*, rice, and bread wheat (Figure 2). All GR proteins are divided into two well-differentiated clades. Clade I contained AsGR1, AlGR1, AiGR1, AtGR1, OsGR2, and TaGR2 proteins, and Clade II consisted of AsGR2, AlGR2, AiGR2, AtGR2, OsGR1, OsGR3, and TaGR1 proteins. Both *A. sativa* and bread wheat are allohexaploid and have the same number of GRs, but there are three AsGRs and four TaGRs in Clades I, while four AsGRs and three TaGRs in Clade II. The distribution and classification of the GR members from A and D subgenomes are identical in the two species, but the classification is different in the unique B or C subgenome. AsGR2-C1 and AsGR2-C2 with close proximity on chromosome 4C were in the same clade, which indicated that AsGR2-C1 and AsGR2-C2 may be a result of expansion of tandem replication.

Additionally, the grouping of AsGR proteins is highly associated with their in silico subcellular localization prediction results. AsGR1 proteins were predicted to be localized in the cytosol, and AsGR2 proteins were mainly localized in the plastid and mitochondria (Table 2). The results are consistent with the experimental subcellular localization of AtGRs and OsGRs and in silico prediction of TaGRs. Taken together, phylogenetic analysis and in silico subcellular localization indicated that GR, as an important oxidoreductase, has formed two main isoforms that may play specific roles in different tissues and under different environmental conditions. Therefore, it is of great significance to precisely regulate cellular GSH pool during plant development and stress conditions.

### 2.4. Motif Pattern and Gene Structure Analysis of AsGR Members

**Figure 3:**
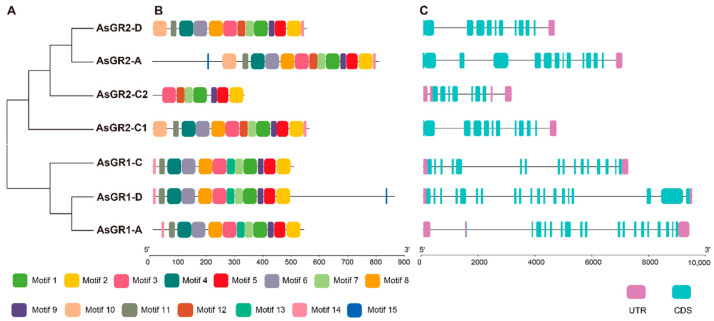
The motif pattern and gene structure of GR members in *A. sativa*. (**A**) Phylogenetic clustering showed that AsGR members were grouped into two subfamilies. (**B**) The schematics represents the motif patterns of AsGR proteins. Different colored boxes indicated the fifteen different MEME-motifs. (**C**) Gene structures of the seven *AsGR* genes. Pink boxes indicate untranslated regions (UTR), and exons and introns are indicated by turquoise boxes and black lines, respectively.

A total of 15 conserved motifs were detected in 7 AsGR proteins, of which motif 1, 2, 3, 5, 7, and 9 were shared by all AsGRs. AsGR2-C2 has the minimum amino acid sequence length and motif number, which is different from the other three AsGR2 proteins. Motif 12 is specific to AsGR2 and is replaced by motif 13 in AsGR1. In addition to AsGR2-C2, motif 10 is also unique to AsGR2. Motif 14 is common in AsGRs, but it is at the N- and C- terminals in AsGR1 and AsGR2, respectively. Motif 15 exists in only two members (at the C-terminus of AsGR1-D, and at the N-terminus of AsGR2-A (Figure 3A,B).

Gene structure analysis showed that most genes had multiple exons and introns. The number of exons of *AsGR1* genes is more than that of *AsGR2* genes, and the maximum number is 19 in *AsGR1-D*. The longest exon is also present in *AsGR1-D* at the 3′ end of the gene. Although the motif distribution pattern of GR protein is basically the same, the structure of most *AsGR* genes is different. *AsGR2-C1* and *AsGR2-D* have similar gene structure and motif distribution, indicating that they may have the same ancestors and similar functions (Figure 3C).

### 2.5. Structural Analysis and Homology Modelling of AsGR Members

**Figure 4:**
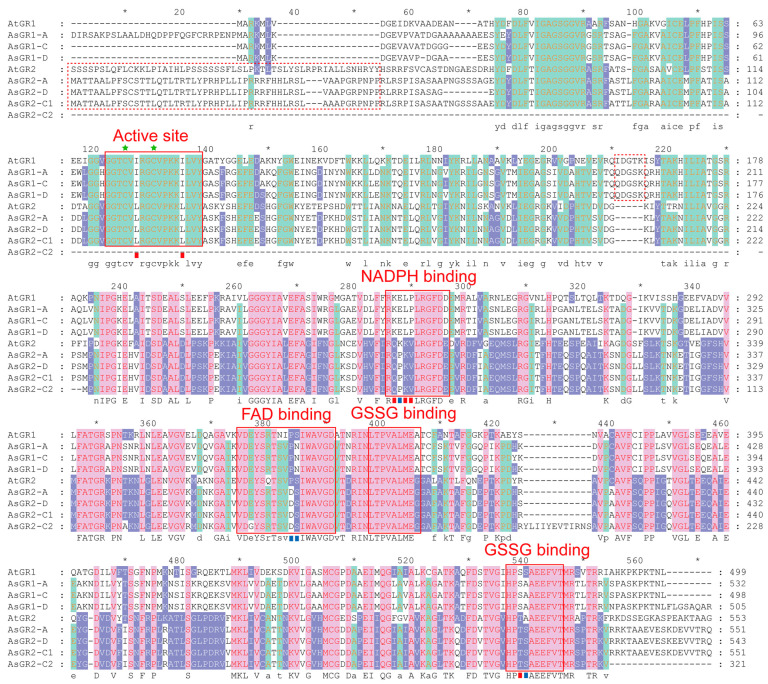
Multiple sequence alignments of AsGR and AtGR proteins. The active site domain, NADPH-binding domain, FAD binding domain, and GSSG binding domain in GR proteins are indicated with a red solid box. The targeting signal peptide for chloroplastic and cytosolic isoforms are showed in red dotted box. Green stars show the cysteine residues involved in catalytic site. Red squares highlight the amino acid substitutions in the GR specific structural domains of cytosolic and chloroplastic GRs. Blue squares highlight the amino acid substitutions in the GR specific structural domains of in AsGRs only.

Multiple sequence alignment of GR protein in *A. sativa* and *Arabidopsis* showed that both AsGR1 and AsGR2 members had highly conserved reactive disulfide bridge domain, NADPH binding domain, FAD binding domain and GSSG binding domain (Figure 4). The conserved disulphide bridge domain containing signature (GGTCV[I/L] RGCVPKK[I/L]LVY) has two active cysteine residues, which participate in the transfer of reducing equivalent from FAD to glutathione. In NADPH binding site, two conserved arginine residues were found for NADPH binding. Amino acid substitutions between AsGR1 and AsGR2 were common. Amino acid substitutions between GR1 and GR2 shared in *A. sativa* and *Arabidopsis* were found in active site, NADPH binding site and GSSG binding site, including I, L, K, Q, P, V, S, and T. There are specific substitutions of AsGR1 and AsGR2, with substitutions in the active site (H/V), the NADPH binding site (E/P), the FAD binding site (N/S), and the GSSG binding site (A/S), respectively. But the substitution of S and A in the GSSG binding sites of *Arabidopsis* was just the opposite. In addition, in *Arabidopsis* and *A. sativa*, the N-terminal targeting signal peptide for chloroplastic isoforms are not conserved, but the targeting signal peptide for cytosolic isoforms are relatively conserved ([I/Q]DG[T/S]K) (Figure 4).

**Figure 5:**
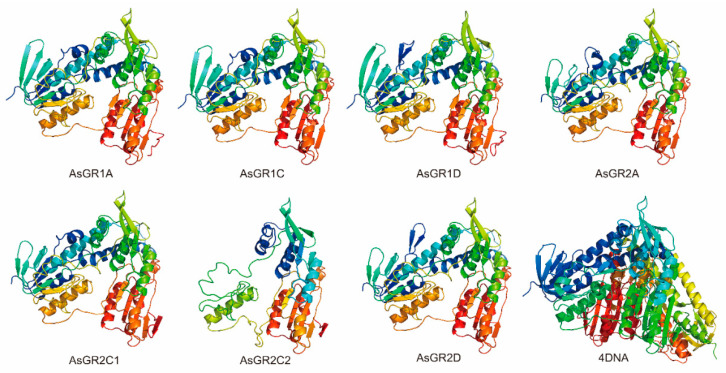
Three-dimensional structure of putative GR proteins from *A. sativa* generated through homology modelling. All AsGR proteins were modelled using the crystal structure of putative glutathione reductase from *Sinorhizobium meliloti* 1021 (PDB: 4DNA) chain A as template.

The three-dimensional structure of seven AsGR family members were modelled using Phyre2 server. Predicted models were based on the reported templates to heuristically maximize the identity and confidence score for the tested sequences. In AsGR proteins, the mainly predicted secondary structures were α-helix (28–33% in each AsGR) and β-strands (23–30% in each AsGR). All AsGR sequences showed high confidence of 100% with the template. In addition, about 46–48% identity was demonstrated between AsGR proteins and the template, suggesting that the predicted AsGRs models had extremely high accuracy (Figure 5).

### 2.6. Identification of Cis-Elements in the Promoter Region of AsGR Genes

**Figure 6:**
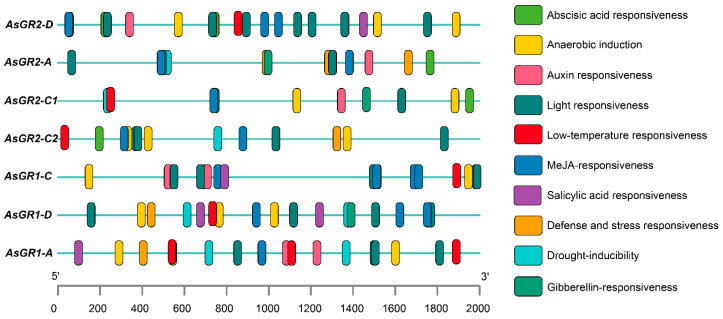
*Cis*-regulatory elements in the promoters of *AsGR* gene family.

The common *cis*-acting element CAAT-box and the core promoter element TATA-box were found to be widely distributed in the promoter region of the *AsGR* genes (data not show). The other *cis*-acting elements are mainly related to hormone responsiveness, anaerobic induction, defense and stress responsiveness and light responsiveness (Figure 6). Hormone responsiveness elements mainly comprised of *cis*-elements associated with ABA, GA, MeJA, SA, and IAA responses. These hormone responsiveness elements appear to be specific for *AsGR1* and *AsGR2*. The stress responsiveness elements are mainly related to drought, low temperature, and defense and stress response. In addition, there are some other elements including MYBHv1 binding site (*AsGR2-D*, *AsGR2-A*, *AsGR2-C2*, *AsGR1-A*, and *AsGR1-C*), zein metabolism regulation (*AsGR2-D*, *AsGR2-A*, *AsGR2-C1*, and *AsGR1-C*), MYB binding site involved in drought-inducibility (*AsGR2-C1*, *AsGR2-A*, *AsGR2-C2*, *AsGR1-A*, and *AsGR1-D*), meristem expression (*AsGR2-C2*, *AsGR1-A*, and *AsGR1-D*), and circadian regulation (*AsGR2-C1*) (Figure 6).

### 2.7. Tissues Specific Expression Analysis of AsGR Genes

**Figure 7:**
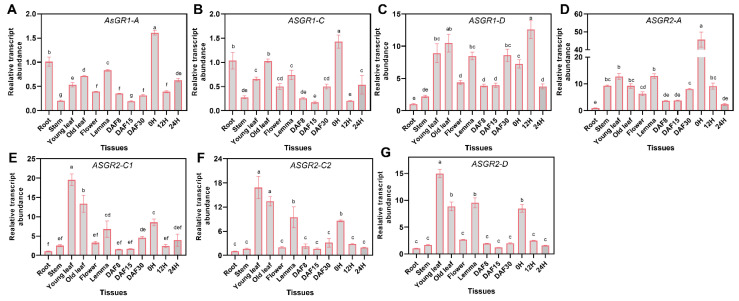
Expression profiling of *AsGR1-A* (**A**)*, AsGR1-C* (**B**)*, AsGR1-D* (**C**), *AsGR2-A* (**D**)*, AsGR2-C1* (**E**)*, AsGR2-C2* (**F**), and *AsGR2-D* (**G**) genes in different tissues of *A. sativa* by qPCR. The relative expression was calculated using transcription level of root as reference. Seeds imbibed for 0 h, 12 h, and 24 h in germination phases were marked as 0 H, 12 H, and 24 H, respectively. Developing seeds at 8, 15, and 30 days after flowering were marked as DAF8, DAF15, and DAF30, respectively. The lower-case letters (a–f) represent statistical significance among different tissues and the vertical bars represent the ±SEM at *p* < 0.05 level for three replicates. The mean values sharing same letters, obtained from Duncan test, are not different significantly.

The expression of *AsGR1* and *AsGR2* genes varied considerably in different tissues (Figure 7). The expression pattern of *AsGR1-A* and *AsGR1-C* was basically identical, and significantly different from that of *AsGR1-D*. *AsGR1-A* and *AsGR1-C* were highly expressed in dry seeds, roots, old leaves, and lemmas, while *AsGR1-D* was highly expressed in dry seeds, leaves, lemmas, and late developing seeds but was with low level in roots. The expression patterns of *AsGR2-C1*, *AsGR2-C2,* and *AsGR2-D* are generally consistent, which showed the highest expression level in young leaves, followed by old leaves, lemmas and dry seeds, and the lowest in roots. The expression of *AsGR2-A* was the highest in dry seeds, followed by leaves and lemmas. On the whole, *AsGR* genes have relatively high expression in leaves and dry seeds, indicating that they may play a role in maintaining leaf function and seed vigor (Figure 7).

### 2.8. Expression Analysis of AsGR1 Genes in Seed Imbibition under Stress

**Figure 8:**
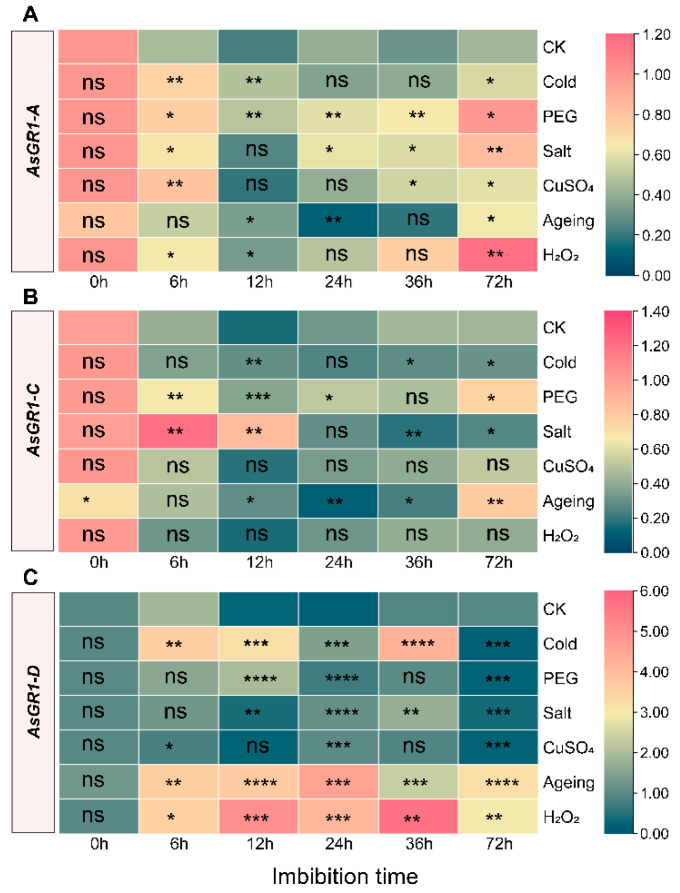
Expression profiling of *AsGR1-A* (**A**), *AsGR1-C* (**B**), and *AsGR1-D* (**C**) during seed imbibition under stress by qPCR. The relative expression was calculated using transcription level of dry seed (0 h) of CK as reference. The significant expression change in comparison to normal conditions (CK) has been calculated using Student’s *t* test. * indicates a significant difference at *p* < 0.05; ** indicates a significant difference at *p* < 0.01; *** indicates a significant difference at *p* < 0.001; **** indicates a significant difference at *p* < 0.0001; ns represents not significant.

Under the non-stress and stress treatments, *AsGR1-A* and *AsGR1-C* showed firstly decreasing and then increasing expression trends, while *AsGR1-D* mainly presented two expression peaks during imbibition (0–72 h). The expression of *AsGR1-C* in aged seeds was significantly down-regulated in dry seeds (*p* < 0.05), which may cause the destruction of redox balance in the early imbibition of *A. sativa* seeds. *AsGR1-A* expression was up-regulated at most imbibition time under stress compared with the control. After 6 h of imbibition, the expression of *AsGR1-A* significantly increased in cold, PEG, salt, CuSO_4_ and H_2_O_2_ treatments. Under PEG and salt treatments, *AsGR1-A* expression increased significantly after 24–72 h imbibition. Under H_2_O_2_ treatment, the expression of *AsGR1-A* was the highest in dry seeds and seeds treated for 36 and 72 h. The expression of *AsGR1-A* in aged seeds was lower compared with other treatments at 24 h imbibition (Figure 8A). *AsGR1-C* showed a minor response to CuSO_4_ and H_2_O_2_ treatments, while it strongly responded to salt treatment at the early imbibition stage (6 h and 12 h). After 72 h imbibition, the expression of *AsGR1-C* was also significantly induced under PEG stress (*p* < 0.05) and in aged seeds (*p* < 0.01) (Figure 8B). *AsGR1-D* was significantly changed after all of the treatments. It was highly up-regulated under H_2_O_2_ and D30 treatment during imbibition (6–72 h). The highest expression level was present at 36 h under H_2_O_2_ treatment. Cold treatment also could induce the expression of *AsGR1-D* at 6–36 h. However, the expression of *AsGR1-D* was lower at 72 h imbibition under cold, PEG, salt and CuSO_4_ treatments than control (Figure 8C). In addition, the expression level of *AsGR1-A* and *AsGR1-D* in aged non-imbibition seeds was not significantly changed (Figure 8A,C).

### 2.9. Expression Analysis of AsGR2 Genes in Seed Imbibition under Stress

**Figure 9:**
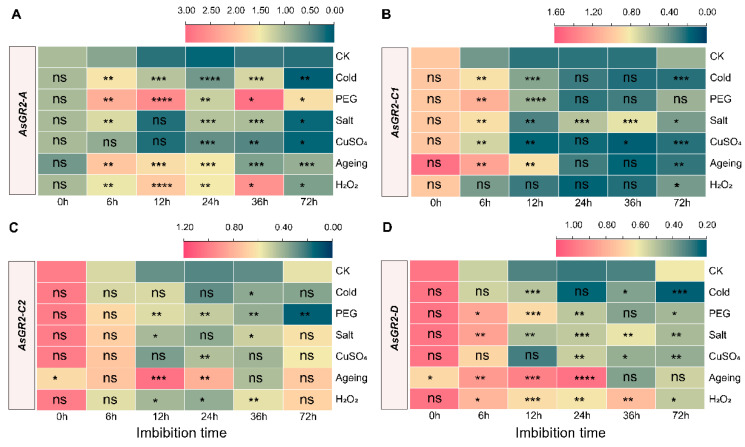
Expression profiling of *AsGR2-A* (**A**), *AsGR2-C1* (**B**), *AsGR2-C2* (**C**), and *AsGR2D* (**D**) during seed imbibition of *A. sativa* under stress by qPCR. The relative expression was calculated using transcription level of dry seed of CK as reference. The significant expression change in comparison to normal conditions (CK) has been calculated using Student’s *t* test. * indicates a significant difference at *p* < 0.05; ** indicates a significant difference at *p* < 0.01; *** indicates a significant difference at *p* < 0.001; **** indicates a significant difference at *p* < 0.0001; ns represents not significant.

The response of *AsGR2* genes to different stress treatments is also different, and most of them have strong response in the early imbibition stage, except for *AsGR2-C2* (Figure 9). After ageing treatment, *AsGR2-C2* and *AsGR2-D* were significantly down-regulated compared with control in non-imbibition seeds (*p* < 0.05) (Figure 9). *AsGR2-A* was considerably induced by various stresses. In detail, among the treatments, PEG, H_2_O_2_ and ageing treatments significantly induced the expression of *AsGR2-A* gene within 72 h of imbibition. The expression of *AsGR2-A* was up-regulated during 6–36 h imbibition, but was down-regulated at 72 h. Under CuSO_4_ and salt treatment, *AsGR2-A* was also significantly changed during 24–72 h (Figure 9A). *AsGR2-C1* was significantly induced for high expression in the beginning of seed imbibition (6 h) under stress treatments, especially PEG and D30 treatments. After 12 h imbibition, *AsGR2-C1* abundance in aged seeds was still in a higher level (Figure 9B). During the imbibition under non-stress and stress conditions, *AsGR2-C2* abundance decreased firstly and then increased. Ageing treatment markedly changed the expression patterns of *AsGR2-C2* during imbibition. *AsGR2-C2* levels in aged seeds was higher than that of the control during 12–24 h. Under PEG treatments, *AsGR2-C2* was up-regulated during 12–36 h, but it was significantly down-regulated at 72 h (Figure 9C). *AsGR2-D* significantly responded to most of the stresses, especially under PEG, salt, ageing and H_2_O_2_ treatments. *AsGR2-D* expression was strongly induced in aged seeds after 6–24 h imbibition. In addition, *AsGR2-D* expression was induced by CuSO_4_ at 24 h and 36 h. However, the abundance of *A_S_GR2-D* under cold, PEG, salt, CuSO_4_, and H_2_O_2_ treatments at 72 h was significantly lower (Figure 9D).

## 3. Discussion

Abiotic stresses, such as drought, salt, cold, heavy metals, and seed ageing, severely affect seed emergence rate and seedling uniformity after sowing. Under adverse conditions, seed germination and seedling growth are inhibited due to the oxidative damage of biomolecules and organelle structures [13,28,29,30]. In addition, seed ageing, which happens inevitably during seed storage, was also found to be mainly caused by accumulating of excessive ROS [31,32]. GSH plays a crucial role in controlling cellular redox homeostasis, and participates in the elimination of excess ROS under adverse conditions. It is indispensable for the earliest metabolic events during seed germination and maintenance of seed vigor [14]. The level of GSH was suggested to be a promising indicator for seed germination and seedling growth, and was correlated with seed longevity [33,34]. GR is considered to be a crucial enzyme that works on the catalytic reduction of GSSG to the reduced GSH form, thereby involving in plant stress resistance, seed vigor maintenance, growth, and development. Genome-wide identification of the GR proteins has been carried out in *Arabidopsis*, rice, wheat, *B. distachyon*, sorghum, Chinese cabbage and other species, and GRs were divided into cytoplasmic isoform and chloroplast/mitochondrial double-localization isoform. Loss of the two types of GR could decrease plant resistance to adversity [14,35,36], and stresses could also induce *GR* gene expression and increase GR activity [16,17,37]. However, GR family members in *A. sativa* have not been identified and analyzed systematically. In this study, we identified *A. sativa* GR family members from the whole genome of oat and analyzed their expression patterns during germination under stress. These gene candidates are useful for the following researches on oat stress resistance breeding and seed vigor improvement.

A total of seven GR proteins were identified in *A. sativa*, which is the same number as that in bread wheat and more than those in other species. It suggested that the GR family members are closely related to their genome duplication events and genome size [24]. GR proteins were initially classified into cytosolic and chloroplast types based on the presence of a typical chloroplast signal peptide at the N-terminus. The AsGR family members were also divided into two clades and were consistent with the predicted subcellular localization. AsGR2-C2 in the chloroplast clade showed a deletion of 230 amino acids at the N-terminus, which may affect its localization and functions. Moreover, GR3 isoform was found in rice, *B. distachyon*, and sorghum, but does not appear in *A. sativa* and bread wheat [24,37]. It suggested that the evolution of *GR* genes in monocots is complicated. Additionally, *AtGR1* and *AtGR2* of *Arabidopsis* have several homologous in *A. sativa*, wheat and rice, which suggests that the evolutionary divergence of *GR* genes occurs after the split of monocotyledon and dicotyledon [38].

To understand the structure consistence and divergence of GR proteins, the gene structures and functional motifs of AsGR members were analyzed. The exon-intron patterns of *AsGR* genes showed that chloroplastic *GR* genes consisted of fewer exons as compared to cytosolic *GR* genes, which was highly consistent with the those of *T. aestivum*, *Ae. tauschii*, *B. distachyon*, *S. bicolor*, *B. juncea*, *B. rapa*, etc. [17,24]. However, the exon number of *AsGR* genes were more than those in most of the other species, which might be due to exon gain, insertion, and exonization during plant evolution [39]. Previous studies have indicated the higher number of exon/intron of cytosolic *GR* genes might be associated with its expression level [17]. Introns also have been shown to increase transcriptional efficiency of numerous genes in a variety of organisms [40]. Moreover, genes with multiple exons and introns can form more variable splicing isoforms and participate in different regulatory networks. Briefly, the structural changes of *GR* genes in oat play an important role in its subcellular expression and transcriptional regulation to cope with adverse environment. A total of 15 motifs were discovered in 7 AsGR proteins, which was consistent with bread wheat and *B. distachyon* [24], but less than 19 motifs in Chinese cabbage, *Brassica rapa*, and *Brassica napus* [16,17]. In most cases, chloroplastic GRs showed more motifs than cytosolic isoforms, and several specific motifs can be found in chloroplastic GRs [24]. In the present investigation, motif 10 associated with the N-terminal targeting signal peptide was found in AsGR2-A, AsGR2-C1 and AsGR2-D and showed high identify with chloroplastic signal peptide of TaGRs and OsGRs [15,24]. Previous studies have found that alanine was the most abundant amino acid in monocot transit peptides, whereas serine was the most abundant in eudicot genotypes. In eudicots, serine was more abundant with 30% more serine on average compared with monocot transit peptides [41]. The serine ratio of chloroplast signal peptide in AsGR proteins was 5.9%, which was significantly lower than that of *Arabidopsis* (27.9%). Additionally, motif 12 and motif 13 are specific to AsGR2 and AsGR1, respectively. Motif 14 is at the N- and C- terminals in AsGR1 and AsGR2, respectively. This divergence might be associated with their biochemical activity and functional diversity, as cytosolic, chloroplastic, and mitochondrial GR isoforms showed great difference in catalytic activity [23,42].

Expression profiling is important for understanding gene functions and screening of candidate genes. Genes specifically expressed in different tissues often have specific functions as well. *AsGR1-A* and *AsGR1-C* were highly expressed in non-imbibed seeds, roots, and leaves, and might have a pivotal role in oxidative stress in these tissues. Similarly, the cytosolically localized BcGR1.1 in Chinese cabbage was highly expressed in the roots, and BcGR1.1-overexpressing *Arabidopsis* showed less damage to the root system under copper stress [16]. The cytosolic isoforms in bread wheat (*TaGR2-A*, *TaGR2-B1*, *TaGR2-B2* and *TaGR2-D*), rice (*OsGR2*) and *Arabidopsis* (*AtGR1*) were also highly expressed in root [15,24]. On the other hand, chloroplast/mitochondrial double-localization *AsGR2* isoforms were expressed strongly in leaves, lemmas, and dry seeds. Chloroplastic GRs has been confirmed to be highly expressed in leaves and have the function of maintaining leaf function and coping with senescence [22]. As lemmas were reported to contribute on seed filling especially under adverse conditions, high expression of GR may act on the protection of photosynthetic system in lemmas [43]. In plants, mitochondria and chloroplasts are the main source of ROS. At the later stage of seed development, seeds are subjected to dehydration stress and may have the risk of excessive accumulation of ROS [44]. *TaGR1-B* and *TaGR1-A* were found upregulated at this stage, which would be conducive to alleviating the excessive accumulation of ROS in seeds. High expression levels of *AsGR2* genes were detected in dry *A. sativa* seeds, which might be beneficial for maintaining seed longevity.

The positive role of *GR* genes in coping with stress has been widely reported. The deletion and low expression of *GR* genes are often associated with poor stress resistance, while overexpression of *GR* improves the capacity to withstand oxidative damage caused by abiotic stresses [16,20,23,45]. Expression profiling indicated *AsGR* genes were significantly induced during seed germination under drought stress, especially in the early stage of imbibition, and *AsGR2* genes were more remarkable. This may be considerable for the special biological process of seed germination, since highly reduced GSH pools are needed in the early stage of seed imbibition, so as to alleviate the accumulation of ROS under strong respiration and display the reduction modification of proteins required for germination [11,14]. *AsGR* genes showed diverse responses to salt treatment during germination. The functions of *GR* genes under salt stress have been well characterized in wheat, tomato, rice, *Stipa purpurea*, and other species [24,36,37,46]. It was reported that salt stress inhibited radicle elongation during seed germination of *A. sativa*, wheat, and rice, while cytoplasmic GR isoforms are highly expressed in roots, which may have a significant protective effect on radicle elongation [47,48]. Under H_2_O_2_ treatment, *AsGR1-A*, *AsGR1-D*, *AsGR2-A*, and *AsGR2-D* expressions were strongly induced during seed imbibition, indicating that they have more significant effects on protection against oxidative damage, and stress specific induction may exist for *AsGR* genes. Seed ageing is an inevitable and complex process, and its mechanisms have not been well revealed so far. The deletion of chloroplast specific GR2 leads to the embryo lethality, while lack of mitochondrial GR2 leads to a partially oxidized glutathione pool and decreased seed ageing resistance of *Arabidopsis* [14,21]. In addition, our previous study found that the expression of *GR1* in aged *A. sativa* seed embryos (aged from 24 d to 42 d) decreased by 50%–70% compared with the control after imbibition for 24 h and that *GR2* levels decreased significantly in minorly and moderately aged seeds, but were raised significantly in severely aged seeds [33]. In present study, the expression of *AsGR2-C2* and *AsGR2-D* decreased significantly in dry seeds after ageing treatment, but they increased rapidly in the early stage of imbibition. Ageing may cause damage to the mitochondrial structural and functional systems [13,30,49]. In the early stage of seed imbibition, excessive respiration can increase the electron transport of mitochondria, which will lead to electron leakage and ROS accumulation [6]. In this study, the expression of *AsGR2-A*, *AsGR2-C1*, and *AsGR2-D* was highly expressed in the early stage of seed imbibition, which could alleviate the damaged redox homeostasis in mitochondria and ensure the energy supply for seed germination. It is suggested that *AsGR2* might be more active on the maintenance of redox homeostasis during germination, especially under PEG, ageing, and H_2_O_2_ stress.

*Cis*-regulatory elements in the promoters control development and physiology by regulating gene expression. Hormone and stress responsive elements are the main functional factors detected in the promoters of *AsGR* genes. This study analyzed the expression of *AsGR* genes under stress during seed germination, but the response of *AsGR* genes to hormone should also be concerned. The crosstalk of hormonal and redox signals has been demonstrated to play a pivotal role in the regulation of plant resistance, leaf senescence, seed germination, seed development, and seed longevity [50,51,52]. Plant growth and environmental adaptation are regulated by various combinations of *cis*-elements, and only a small set of responsive genes can be regulated by a single cis-element [53]. GA, ABA, SA, MeJA, and IAA responsive elements combined with stress responsive elements may play an important role in the regulation of stress resistance and development. Moreover, these *cis*-elements seem to be species-specific and subcellular localization-specific. For instance, ABA-responsive elements were not found in the promoter of pea *GR* genes [54], but only in the promoter of *AsGR2* genes and not in the promoter of *AsGR1* genes in *A. sativa*. ABA is a crucial factor of seed vigor, and it has been found to play a regulatory role in mitochondrial ROS production and energy metabolism in recent years [55,56]. It is hypothesized that the interaction between mitochondrial GR2 and ABA may play a role in regulating seed vigor. Consequently, the regulatory relationship between *AsGR2* genes and hormone signaling should be focused on in the future.

## 4. Materials and Methods

### 4.1. Identification and Chromosomal Location of AsGR Genes

The genome information of *A. sativa* and its possible ancestors *A. longiglumis* and *A. insularis* were downloaded from the GrainGenes database (https://wheat.pw.usda.gov/GG3/, accessed on 24 November 2021). The rice GR proteins (LOC_Os02g56850.1, LOC_Os03g06740.1 and LOC_Os10g28000.1) and *Arabidopsis* GR proteins (AT3G24170 and AT3G54660) were used as query sequences to obtain possible GR proteins in three genomes by BlastP search with a cutoff e-value of 1 × e^−5^. Further, the predicted GR protein sequences were reconfirmed by Simple Modular Architecture Research Tool (SMART; http://smart.embl-heidelberg.de/smart/batch.pl, accessed on 26 February 2022), Pfam (http://pfam.xfam.org/, accessed on 26 February 2022), and NCBI conserved domain database (https://www.ncbi.nlm.nih.gov/, accessed on 26 February 2022) [24,57]. Chromosomal location of oat GR members was displayed by TBtools. For nomenclature, *AsGR* genes were named according to wheat GR genes, which referenced the international rule for wheat gene symbolization (http://wheat.pw.usda.gov/ggpages/wgc/98/Intro.htm, accessed on 16 February 2022) and presented the chromosomal information of *GR* members.

### 4.2. Physiochemical Properties and Subcellular Localization

The physical and chemical properties of *A. sativa* GR proteins were analyzed by using the online program ExPaSy-ProtParam (https://web.expasy.org/protparam/, accessed on 16 March 2022), including amino acids (AA) number, molecular weight (MW), theoretical isoelectric points (pI), etc. Protein subcellular localization prediction was performed using the online tools WoLF PSORT (https://wolfpsort.hgc.jp/, accessed on 28 March 2022), CELLO v.2.5 (http://cello.life.nctu.edu.tw/, accessed on 28 March 2022), and Cell-PLoc 2.0 (http://www.csbio.sjtu.edu.cn/bioinf/Cell-PLoc-2/, accessed on 28 March 2022).

### 4.3. Phylogenetic Analysis of GR Proteins of Oat and Other Plant Species

The identified GR proteins were used to conduct multiple sequence alignment with GR members derived from *Arabidopsis*, rice and bread wheat by ClustalX2 software with the default parameters [58]. Phylogenetic tree was constructed using the sequence alignment results with the neighbor-joining (NJ) method in MEGA6.0 [59]. The bootstrap was tested 10,000 times and expressed as a percentage of 1000 replicates.

### 4.4. Motif Pattern and Gene Structure Analysis of AsGR Members

GR protein sequences were used to predict the occurrence of functional motifs in the MEME website (http://meme-suite.org/, accessed on 19 February 2022). TBtools ver 1.0986 were used to edit the motif pattern and gene structure diagrams [60].

### 4.5. Multiple Sequence Alignment and Homology Modelling of AsGR Proteins

Multiple sequence alignment of AsGRs with already known GRs from *Arabidopsis thaliana* (AtGR1 and AtGR2), was performed using ClustalX2 software and visualized using GeneDoc software [58]. The conserved domain and targeting signal peptides which are essential in redox activity and subcellular localization are boxed according to previous description [17,23]. All of the AsGR members were three-dimensionally modeled using the Phyre2 server (http://www.sbg.bio.ic.ac.uk/phyre2/html/, accessed on 2 April 2022) [61]. The topmost hit crystal structure of putative glutathione reductase from *Sinorhizobium meliloti* 1021 (PDB: 4DNA) chain A was used as a template.

### 4.6. Identification of Cis-Elements in the Promoter Region of AsGR Genes

In order to determine the responses of *AsGR* genes to stress treatments, we retrieved 2 kb of the deduced promoter region from *A. sativa* genome database to identify the *cis*-regulatory elements using PlantCARE [62].

### 4.7. Plant Material, Treatment and Collection of Tissues

Oat (cv Challenger) seeds with germination percentage of 100% were used in the present study. For *A. sativa* tissue-specific expression profiling of *AsGR*s, twelve samples including seeds, roots, leaves, stems, florets and lemmas were used. In detail, seed samples were collected in imbibition phases (imbibition for 0 h, 12 h and 24 h) and development phases (8, 15 and 30 days after flowering, DAF). Young leaves, lemmas and florets were collected at flowering stage, and old leaves were from plants at 30 DAF. The roots were obtained from 10-day-old plants. To understand the expression profiling of *AsGR*s during seed germination under salt, drought and heavy metal stresses, seeds were subjected to 150 mM NaCl, 20% PEG 6000 and 200 mg L^−1^ CuSO_4_·5H_2_O solution for imbibition, respectively [63,64,65,66]. For oxidative stress, seeds were imbibed in 2% H_2_O_2_, since the germination percentage decreased from almost 100% to 45% after treatment. For control, cold treatment and ageing treatment, seeds were imbibed in distilled water. Seed ageing treatment were according to Xia et al. [13], and seeds ageing time was according to our previous investigation [33]. Cold treatment was carried out at 10 °C, while control and other treatments were imbibed at 20 °C in the plant growth incubator under 16 h dark and 8 h light cycle. Seed samples were collected after imbibition for 0 h, 6 h, 12 h, 24 h, 36 h and 72 h, which included the three major phases of oat seed imbibition under normal conditions. Each tissue sample contained three biological replicates, and each replicate was collected from at least ten seeds or plants. The collected tissue samples were then ground into powder in liquid nitrogen.

### 4.8. qRT-PCR and Statistical Analyses of AsGR Genes

Total RNA was extracted using Quick RNA isolation Kit (Huayueyang Biotech Co., Ltd., Beijing, China). First-strand cDNA was synthesized from 1 μg RNA using *EasyScript*^®^ All-in-One First-Strand cDNA Synthesis SuperMix for qPCR Kit (TransGen Biotech, Beijing, China). qRT-PCR analysis was performed on a CFX96 Real-Time System (Bio-Rad, Hercules, CA, USA) using 2×RealStar Green Fast Mixture (Genstar, China) with *AsEIF4A* gene as reference gene [67]. The thermal cycle program was 95 °C for 25 min, and 40 cycles of 95 °C for 5 s and 60 °C for 10 s. The comparative delta-Ct method was used to calculate the relative transcript levels of *AsGR* genes [68]. The comparison of relative expression level between different tissues were performed in SPSS Statistics 22 using ANOVA and a Duncan’s test, and visualized using GraphPad Prism version 8.0. The expression heatmaps of *AsGR* genes during seed imbibition under stress were visualized using TBtools ver 1.0986. The significant expression change in comparison to normal conditions was calculated using Student’s *t* test. The details of the primers used in qRT-PCR assay are listed in Table S1.

## 5. Conclusions

In summary, seven *AsGR* genes were identified and mapped on six chromosomes of A, C and D subgenomes. Phylogenetic analysis and subcellular localization of AsGR proteins suggested they were divided in two sub-families, AsGR1 and AsGR2, which showed predicted subcellular location of cytoplasm and chloroplast/mitochondrion, respectively. Structure analysis suggested *AsGR* genes were highly conserved and that *AsGR1* contained higher number of exon/introns. Both *AsGR1* and *AsGR2* genes have relatively high expression in leaves and dry seeds, suggesting they may play a role in maintaining leaf function and seed vigor. Their responses to drought, salt, cold, copper, H_2_O_2_, and ageing treatments were quite different during seed imbibition. Among the seven *AsGR* genes, *AsGR1-A, AsGR1-C, AsGR2-A,* and *AsGR2-D* responded more significantly, especially under drought, ageing, and H_2_O_2_ stress. It suggested that they might be more active on the maintenance of redox homeostasis during seed imbibition under unfavorable conditions. The present study will lay the foundation for future studies on verifications of the precise regulation of *GR* genes in *A. sativa*. Finally, stress-responsive *AsGR* genes can be used for genetic improvement of stress resistance and seed vigor in oat.

## Figures and Tables

**Table 1:** Physico-chemical properties of GR family in *A. sativa*.

Gene Names	Gene ID	Chromosomal Location	Number of Amino Acids	Molecular Weight	pI	Instability Index	Negatively/Positively Charged Residues
*AsGR1-A*	AVESA.00001b.r1. 6Ag0001934.1	6A: 328352411.. 328361762	533	57.176	5.96	28.52	66/60
*AsGR1-C*	AVESA.00001b.r1. 6Cg0000969.1	6C: 102116290.. 102123495	498	53.430	5.82	25.74	62/56
*AsGR1-D*	AVESA.00001b.r1. 6Dg0001672.1	6D: 288925140.. 288934590	853	93.097	6.00	29.34	115/105
*AsGR2-A*	AVESA.00001b.r1. 4Ag0002233.1	4A: 337954968.. 337961977	798	87.477	9.59	53.87	90/123
*AsGR2-C1*	AVESA.00001b.r1. 4Cg0001182.1	4C: 94048476.. 94053156	551	59.450	6.93	40.76	62/61
*AsGR2-C2*	AVESA.00001b.r1. 4Cg0001191.1	4C: 95512612.. 95515711	321	34.718	5.34	35.01	41/32
*AsGR2-D*	AVESA.00001b.r1. 1Dg0003199.1	1D: 455767235.. 455771855	543	58.772	6.93	42.23	62/61

**Table 2:** In silico subcellular localization prediction of GR proteins.

Species	Proteins	CELLO	WoLF PSORT	Plant-mPLoc
*A. sativa*	AsGR1-A	Cytoplasm	Cytoplasm	Chloroplast. Cytoplasm
	AsGR1-C	Cytoplasm	Cytoplasm	Chloroplast. Cytoplasm
	AsGR1-D	Cytoplasm	Cytoplasm	Chloroplast. Cytoplasm
	AsGR2-A	Mitochondrion	Chloroplast	Chloroplast. Cytoplasm. Mitochondrion
	AsGR2-C1	Chloroplast	Chloroplast	Chloroplast. Cytoplasm. Mitochondrion
	AsGR2-C2	Cytoplasm	Cytoskeleton	Chloroplast. Cytoplasm. Mitochondrion
	AsGR2-D	Chloroplast	Chloroplast	Chloroplast. Cytoplasm. Mitochondrion
*A. longiglumis*	AlGR1-A	Cytoplasm	Cytoplasm	Chloroplast. Cytoplasm
	AlGR2-A	Chloroplast	Chloroplast	Chloroplast. Cytoplasm. Mitochondrion
*A. insularis*	AiGR1-C	Cytoplasm	Cytoplasm	Chloroplast. Cytoplasm
	AiGR1-D	Cytoplasm	Cytoplasm	Chloroplast. Cytoplasm
	AiGR2-C	Chloroplast	Chloroplast	Chloroplast. Cytoplasm. Mitochondrion
	AiGR2-D	Chloroplast	Chloroplast	Chloroplast. Cytoplasm. Mitochondrion

## Data Availability

Data is contained within the article and supplementary material.

## Supplementary Materials

The following supporting information can be downloaded at: https://www.mdpi.com/article/10.3390/ijms231911650/s1.
