# Supplementary material for: Genome-Wide Analysis and Expression Profiling of Glutathione Reductase Gene Family in Oat (Avena sativa) Indicate Their Responses to Abiotic Stress during Seed Imbibition
Source: Int J Mol Sci. 2022 Oct 1;23(19):11650. doi: 10.3390/ijms231911650 (PMC9569478; doi:10.3390/ijms231911650)
Supplement: Supplementary file 1 [file ijms-23-11650-s001.zip › Table S1 Primer information of AsGR genes and reference gene for qPCR analysis.pdf]

Table S1. Primer information of *AsGR* genes and reference gene for qPCR analysis

No.	Gene name	Primer sequence (5′-3′)	Product size (bp)	Tm (°C)
1	*AsGR1A*	F: ATCAACGGGGACATCAACTACAAC	111	59.0
		R: CGTCACGCCAGAATTACCAAGG		59.3
2	*AsGR1C*	F: GCAACTGGTAGCCGAGCAC	165	58.1
		R: TTCAGCACCCAACCCTCTCC		58.7
3	*AsGR1D*	F: TTAGCGATACCGAGATGAAGGATG	99	58.0
		R: CGTACCAATACCATGAACAGAAGC		57.9
4	*AsGR2A*	F: CCGTCGCCGTCGTCACTG	199	59.9
		R: CCATCCTTCTCCTTCTCCTTCTCC		59.6
5	*AsGR2C1*	F: CGGCGGAGTACGACTACGAC	83	59.4
		R: GAAGAGGGTGGTGGCAAAGC		58.5
6	*AsGR2C2*	F: TGCCAAATATCCCAGGAATAGAGC	164	45.8
		R: TTGCCGAATGAACACATGAACG		45.5
7	*AsGR2D*	F: GCCGTTGGAGATGTTACTGATAGG	113	58.9
		R: GCACTGTGATCTGGTTTGGTAGG		59.1
8	*AsEIF4A*	F: TCTCGCAGGATACGGATGTCG	88	63.3
		R: TCCATCGCATTGGTCGCTCT		63.6
